# Researcher views on returning results from multi-omics data to research participants: insights from The Molecular Transducers of Physical Activity Consortium (MoTrPAC) Study

**DOI:** 10.1186/s12910-025-01174-9

**Published:** 2025-02-07

**Authors:** Kelly E. Ormond, Caroline Stanclift, Chloe M. Reuter, Jennefer N. Carter, Kathleen E. Murphy, Malene E. Lindholm, Matthew T. Wheeler

**Affiliations:** 1 Department of Genetics, Stanford University School of Medicine, Stanford, CA USA; 2 Center for Inherited Cardiovascular Disease, Division of Cardiovascular Medicine, Department of Medicine, Stanford University School of Medicine, Stanford, CA USA; 3 Center for Undiagnosed Diseases, Stanford University, Stanford, CA USA; 4 Stanford Center for Biomedical Ethics, Stanford University School of Medicine, Stanford, CA USA; 5 Health Ethics and Policy Lab, Dept of Health Sciences and Technology, ETH-Zurich, Zurich, Switzerland

**Keywords:** Multi-omics, Attitudes, Research, Personnel, Informed Consent, Genomics

## Abstract

**Background:**

There is growing consensus in favor of returning individual specific research results that are clinically actionable, valid, and reliable. However, deciding what and how research results should be returned remains a challenge. Researchers are key stakeholders in return of results decision-making and implementation. Multi-omics data contains medically relevant findings that could be considered for return. We sought to understand researchers' views regarding the potential for return of results for multi-omics data from a large, national consortium generating multi-omics data.

**Methods:**

Researchers from the Molecular Transducers of Physical Activity Consortium (MoTrPAC) were recruited for in-depth semi-structured interviews. To assess understanding of potential clinical utility for types of data collected and attitudes towards return of results in multi-omic clinical studies, we devised an interview guide focusing on types of results generated in the study for hypothetical return based on review of the literature and professional expertise of team members. The semi-structured interviews were recorded, transcribed verbatim and co-coded. Thematic trends were identified for reporting.

**Results:**

We interviewed a total of 16 individuals representative of 11 sites and 6 research roles across MoTrPAC. Many respondents expressed positive attitudes regarding hypothetical multi-omics results return, citing participant rights to their data and perception of minimal harm. Ethical and logistical concerns around the return of multi-omics results were raised, and they often mirrored those in the published literature for genomic return of results including: uncertain clinical validity, a lack of expertise to communicate results, and an unclear obligation regarding whether to return multi-omics results. With the exception of privacy concerns, respondents were able to give examples within multi-omics of how each point was relevant. Further, researchers called for more guidance from funding agencies and increased researcher education regarding return of results.

**Conclusion:**

Overall, researchers expressed positive attitudes toward multi-omic return of results in principle, particularly if medically actionable. However, competing ethical considerations, logistical constraints, and need for more external guidance were raised as key implementation concerns. Future studies should consider views and experiences of other relevant stakeholders, specifically clinical genomics professionals and study participants, regarding the clinical utility of multi-omics information and multi-omics results return.

**Supplementary Information:**

The online version contains supplementary material available at 10.1186/s12910-025-01174-9.

## Background

Research on human subjects generates vast amounts of information, some of which holds potential value to participants [1, 2]. However, the primary goal of human subjects research is to develop results to benefit a greater good. Despite a growing consensus of an obligation to return research results to individual study participants that are clinically actionable, valid, and reliable [2–4], deciding what and how results should be returned remains a challenge [5, 6]. Respect for participants’ autonomy (i.e. their rights to their own data), a duty to warn of imminent harm, and a partial-entrustment account must be weighed against potential harm to study participants and burdens on the research enterprise and on participant-volunteers [6, 7]. Further, returning research results raises the possibility of therapeutic misconception, when participants confuse the obligations of research versus clinical care [3, 8]. Therefore, developing return of results protocols for research studies remains a challenge.

In 2010, an NIH National Heart, Lung, and Blood Institute (NHLBI) expert working group on returning *genomic* individual research results specified that results *should* be offered to properly consented study participants when the following criteria are met: (a) the genetic finding confers substantial risk of health implications (b) there are established therapeutic or preventive interventions with potential to change the clinical course of disease (i.e. are actionable) and (c) the test is analytically valid and disclosure follows applicable laws [4]. This report specified only *genomic* research results, and does not reflect recent multi-omic advances [9]. More recently, the National Academies of Sciences, Engineering, and Medicine (NASEM) published a framework [2] to inform the decision to return individual research results from any tests on human biospecimens including evaluation of (1) ‘value to participants’ and (2) ‘feasibility’ on a study-by-study basis. What results researchers are obligated, allowed, or prohibited to return in the current regulatory landscape, particularly those which may not be accurate, clinically actionable, or have clear meaning, remains uncharted territory for multi-omics research. Only a few papers attempt to operationalize return of individual results for multi-omics [10].

Whereas genomic data is limited to DNA, multi-omic data together dynamically characterize DNA modifications (epigenomics), RNA (transcriptomics), proteins (proteomics), and metabolites (metabolomics) at scale [11]. Analysis of these “omes”, either individually or in combination, offer a way to understand biological systems and ‘map’ pathophysiology of human health and disease [12]. Large efforts are underway to generate multi-omic data in rare disease and healthy volunteer cohorts [13–17]. Numerous examples show multi-omics results beyond DNA sequencing can contain medically relevant findings [18–22]. Plasma untargeted proteomics have ability to detect biomarkers of both acute and chronic disease, such as identification of myocardial injury or diabetes mellitus [18]. RNA sequencing (RNA-seq) can reliably identify inherited variants which confer an increased risk for diseases [23], while untargeted metabolomics often measure analytes that are tested in clinical labs to guide clinical care. Multi-omics results may include data generally recommended not to be disclosed (e.g. risk of Alzheimer's disease [19]). Despite growing evidence that multi-omics results may contain valuable and clinically actionable information, there is no specific guidance for returning multi-omics results. Compared to genomic data, multi-omic data are more complex, multi-modal, and dynamic which could complicate the analysis and interpretation [24–26].

Multiple stakeholder groups’ perspectives–researchers, research participants, clinicians, Institutional Review Board (IRB) members, funders, and the public– are important to consider in the return of research results process as new technologies arise. Researchers are a particularly critical voice as they are responsible for decisions to return results, protocol development, and implementation [27]. To minimize potential burdens of that responsibility and any potential moral distress, their views should be considered when developing policy [28]. Multiple studies have surveyed researcher views, attitudes, or perspectives about return of results for genomics. For example, often finding the features of a given condition (severity, treatability, and heritability [29–35], as well as the perception of clinical validity and certainty of the results influence the decision to return [30]. In examining return of results in epigenetics research, the clinical uncertainty of these results was critical [36]. There is limited literature that has discussed the return of non-genomics omics results. This study seeks to understand researchers' views regarding the potential for returning research results from multi-omics data.

## Materials and methods

### Design

We conducted exploratory and in-depth semi-structured interviews with researchers involved with a multi-omics research consortium (MoTrPAC), to assess their views regarding the return of multi-omics research results to study participants. The NIH Common Fund initiative Molecular Transducers of Physical Activity Consortium (MoTrPAC) is an effort to investigate the molecular drivers of exercise adaptation through generation of multi-omic multi-tissue datasets derived from multiple animal and human sub-studies [37–39]. [40]. MoTrPAC includes researchers from many different areas of research expertise, with diverse training, varied experience, and unique perspectives on the study participants, biological sampling, multi-omic data generation and multi-omic results.

### Subjects and recruitment

MoTrPAC researchers were invited to participate in semi-structured interviews via a recruitment email sent to consortium members with a link to a recruitment survey for eligibility and limited demographic information. Individuals with an email address registered with the MoTrPAC coordinating center were considered eligible. Members of this study team were excluded. We employed purposeful sampling to achieve maximum variation amongst interviewees [41], stratifying based upon demographic information (gender, age, human genetics research years of experience), researcher role, and MoTrPAC site.

### Development of the interview guide

An interview guide (Supplemental Methods) was novelly devised from the review of literature cited in the introduction and professional expertise with offering and returning genetic testing results and multi-omic results (CS, CR, JK, KEO, MW) and domain expertise in the MoTrPAC human sub-studies (MW, KEO, ML). The interview guide examined the following domains: (a) understanding of the potential clinical utility for the types of data being collected, (b) knowledge of current plan for return of results in MoTrPAC clinical studies, (c) hypothetical preferences for types of results to be returned in MoTrPAC, and (d) attitudes towards return of multi-omic results. Education regarding hypothetical types of results which could be returned to participants was provided for all researchers regardless of their experience and expertise.

### Interview process/data collection

Semi-structured audio interviews were conducted by Zoom by a single interviewer (CS) between October 2020 and January 2021. Interviews were audio recorded, transcribed verbatim, de-identified, and checked for accuracy and familiarization.

### Data analysis

We used a thematic analysis approach [42]. A codebook was developed by a team (CS, KEM, KEO) based on a literature review and inductive coding from data. This codebook was applied and revised iteratively until consensus was reached after four transcripts. All interview transcripts were then coded by a single researcher (CS) using Dedoose software Version 8.3.45. Every other transcript was co-coded to consensus with a second coder (KEM) to ensure codebook stability and consistency of code application. Coded excerpts were sorted by prevalence and code co-occurrence, then were analyzed for emergent themes. Final themes were agreed upon by consensus of the entire research team. Themes and quotes were selected for prevalence and to show breadth of viewpoints.

## Results

Of the 663 eligible researchers who received the recruitment email, 35 researchers responded to the enrollment survey (5.3%). Of survey respondents, 31 agreed to be interviewed and 16 completed interviews. Table 1 describes the demographics. The interviewees represented 11 of 31 MoTrPAC sites (35%) and held a range of research functions, including both participant-facing (31%) and non participant-facing (69%) roles. Interviews lasted an average of 40 min (range 31–57 min).

**Table 1:** Demographics (*N* = 16)

**Category**	*n* (%)
**Gender**	
Male	7 (44%)
Female	9 (56%)
**Principal Investigator Status**	
Yes	6 (37%)
No	10 (63%)
**Role within MoTrPaC**	
Recruitment / Consent	2 (12.5%)
Exercise Physiologist	2 (12.5%)
Clinician Investigators (MD, MD/PhD)	2 (12.5%)
Scientist Investigators (PhD)	4 (25%)
Researcher Data Production	2 (12.5%)
Researcher Data Analysis	4 (25%)
**Participant Facing Role**	
Yes	5 (31%)
No	11 (69%)
**Years Research Experience**	
1–5	6 (37%)
6–10	4 (25%)
> 10	6 (37%)

Thematic analysis of the data yielded three overarching themes (Table 2): (1) Reasons to support return of individual multi-omics results; (2) Concerns; and (3) The need for guidance from funding agencies and national organizations. It is noteworthy that much of the data we obtained regarding the return of multi-omic research data echoes attitudes that have been published regarding genomics. Thus we will focus below on the data that arises regarding multi-omics specifically, presenting more global data when it serves to explain the participant views about multi-omics or was presented in tandem.

**Table 2:** Thematic analysis yielded 3 overarching themes and relevant sub-themes

Themes	Subthemes
**Theme 1:** Reason to support return of individual multi-omics result	• *Participant Rights to Their Own Data* • *Perception of Minimal Harms* • *Future Value of Multi-omics Information*
**Theme 2:** Concerns about return of multi-omics research result	• *Complex Interpretation with Uncertain Clinical Validity* • *Lack of Clear Obligations to Return Multi-omics Results* • *Expertise Required to Communicate Multi-omics Result*
**Theme 3:** Need for external guidance from funding agencies	

### Reasons to support return of individual multi-omics results

Reasons given by respondents to support return of multi-omic research results were primarily ethical and mirrored the reasons that participants gave to explain why they supported return of more research results in generally, including genomic research results.

#### Participant rights to their own data

Researchers expressed high regard for research participants’ autonomy, stressing the importance of informed consent and the importance of participant preferences. The majority of researchers supported returning multi-omics research results because they believed research participants have a right to the data generated from their individual samples. Researchers described how participants volunteer “their body, their cells” (Researcher #1, Recruitment/Consent) so data should be returned if they chose. For one researcher, making decisions for participants about which data should be returned was paternalistic:

“I don't like when somebody tries to play the role of God and makes decisions for patients or participants like … which data should be released and which data should not be released.”

(Researcher #8, Clinician Investigator)

#### Perception of minimal harms

Some researcher’s support for the return of individual multi-omics results relied on the assumption that there is minimal harm in doing so, especially if an expert trained in communicating complicated medical information returns the results:

“I just think all the concern is way overblown and the benefit of providing individuals data about themselves way outweighs the harm… I just don’t see any harms here”

(Researcher #6, Clinician Investigator)

#### Future value of multi-omics information

Other researchers discussed the benefits of providing individuals with information about themselves that they might not learn or have access to outside of a research context, and the constant advancement of scientific knowledge. Researchers expected information generated in the study with uncertain clinical validity could yield future clinical utility:

“What we've come up with in the last decade and think about what will come up with in the future, in the next decade. … If you could have kind of a catalog of some proteomics or metabolomics information about yourself that at the moment isn't, or may not be that useful, but just have that in your back pocket and years down the road, be able to plug that into . . . new analyses and get new information. That would be awesome”

(Researcher #11, Data Analyst)

Finally, less prominent reasons given in support of returning all research results, including multi-omics, included increased rapport between participants and researchers, promoting altruism, and improved study retention.

### Reasons against returning multi-omics research results

Ethical and logistical concerns were raised regarding return of results for MoTrPAC in general and for multi-omics results.

#### Complex interpretation with uncertain clinical validity

Researchers stressed the complexities of clinical interpretation of multi-omics data. Some of these concerns were primarily logistical –– the “sheer volume of data”, time consuming and expensive scientific workflows to interpret data and provide return of results for a large study cohort;

“I don't know what the final sample size will be. But let's say it's 2000, around 3000 people. How do we provide results, logistically, to that number of people?... I mean, [sic] highly in favor of giving back results but… I don't know how they will manage to do that.”

(Researcher #15, Scientist Investigator)

Others’ concerns were more conceptual, such as the ability to draw accurate conclusions and find clinically actionable information to provide to study participants. The possibility to misinterpret the data led some researchers to suggest multi-omic data could be provided if requested, but without interpretation. While researchers acknowledged the possibility of valuable findings, they stressed the interpretation could be burdensome for the bioinformatics pipeline;

“I don't think most of it's easy to interpret. I think some of it could be… I don't think most of the information is actionable, quite frankly, unless you go in and spend time on it, meaning you have to do outlier analysis to be able to see if somebody really looks like they have a strange pattern for most of the omics information…but I think on the multi-omics side, it's possible things will be uncovered and if they've consented, yes”

(Researcher #13, Scientist Investigator)

#### Lack of clear obligation to return multi-omics results

Multiple researchers expressed they did not feel an ethical obligation or moral duty to return multi-omics results. This was due to the perception of unclear clinical validity or utility of these results with current scientific knowledge. Several researchers explicitly mentioned the obligation to return multi-omic data felt different compared to genomic data, in part because the health implications are less clear for multi-omics and therefore less concerning. Still others alluded to precedent in the field as justification the obligation to return multi-omics results remains vague;

“I wouldn't … have the same kind of moral thoughts of, like, oh, what if we knew something that we didn't tell them about and then it came back to bite them later? …. I could see the proteomics data, like, in particular, what would somebody even do with a bunch of information about their protein expression levels? Is there any sense that they could make out of it? So potentially that information is just not something that's worth returning raw.”

(Researcher #11, Data Analyst)

“I do think that anything where we have clear risk associated with genetic variant, yes. Now for all the other classes of data, the proteomics, etc, then unless it points to a genetic variant, the proteomics, metabolomics, the epigenetics all those are usually soft risks. They're really not established. And nor should I think we worry about those data. Only the genetic data is the domain that I think we need to be concerned about.”

(Researcher #6, Clinician Investigator)

“I'm not aware of people that return RNAseq or mass spec back to people … I'm not sure we owe it to our participants to give them everything and it's not typically done.”

(Researcher #16, Data Analyst)

#### Expertise required to communicate multi-omics results to participants

Finally, many researchers emphasized the need for expertise to translate complex multi-omic data and “package them up in a meaningful way” (Researcher #16, Data Analyst). The ability to answer participant questions about health implications and provide support for follow up care emerged as a key concern. Researchers also worried study participants may bring their multi-omics research results to general practitioners unaffiliated with the study, who would be unable to answer questions about the meaning of the results or make evidence-based recommendations. Overall, they felt returning results without the ability to accurately answer questions would be futile:

“What's going to be returned is something that's potentially important to their health . . . Nobody on my team could answer those questions. I don't think a lot of physicians might necessarily be able to. … I feel very strongly about that because if you just get a piece of paper and you can't clarify what's on it by asking somebody it's almost worthless, frankly.”

(Researcher #10, Scientist Investigator)

Attitudes about communicating with participants were often shaped by their understanding of clinical testing methods and utility. One researcher felt there is a collective lack of knowledge about potential risks of returning multi-omics results. They deeply worried about the ability to warn participants of the potential impact receiving results from the study could have;

"As a clinical scientist my primary concern is making sure that I can adequately inform a research volunteer of the risks associated with returning results. And that has not been an easy thing to do for me in MoTrPAC because of the breadth of the type of results that we could ultimately share with participants. … we don't know what the potential clinical impact of that could be and … most people don't understand how getting that those results could impact them … this has been very challenging for me because I don't know that I feel comfortable in knowing that I can adequately inform participants of what the risks to them might be."

(Researcher #9, Scientist Investigator)

Subjective factors, such as personal or family health-related experiences, professional background, and tolerance for uncertainty, often influenced researchers’ views as well. For example, one researcher with clinical training questioned “am I qualified to give that information?… and if so, once they receive that information, handle them from a psychological standpoint?” (Researcher #2, Recruitment/Consent). While this researcher stressed the ability to clearly communicate the information and the psychological impact research results may have, other researchers did not consider psychological ramifications important: “most of the participants that enter into the study to begin with are relatively mentally capable of stepping over minor anxieties so I don't see that being a contributing challenge” (Researcher #4, Exercise Physiologist). 75% (12/16) of those interviewed thought participants should receive any multi-omics results from a genetic counselor, or at least have access to a genetic counselor. Respondents cited genetic counselors’ expertise in interpreting clinical genomics, delivering complex information, and providing appropriate psychosocial support as part of results disclosure.

In addition to the sub-themes presented above, other concerns raised about return of results in general included privacy and insurance discrimination and financial costs. Interestingly, participants only brought up re-identification in the context of genomics results, not multi-omics results.

“In this day and age of technology, somebody could decipher your genetic code and figure out that information belongs to you. What are those ramifications and are they prepared for that?”

(Researcher #2, Recruitment & Consent)

#### Need for external guidance from funding agencies and national organizations

In the present study, nearly half of respondents (7/16) specifically mentioned the need for more clear guidance from federal agencies to standardize return of results protocols, particularly for multi-omics projects. Researchers reflected on procedural questions such as funding available within a grant cycle, verification of results in CAP / CLIA certified labs, and responsibility for facilitating referrals at disclosure. Many researchers contextualized the problem as “bigger than MoTrPAC”.

“This is something that should be discussed at a much higher level than just MoTrPAC. … NIH and other federal agencies that are financially supporting these studies should actually have a policy that should be vetted by medical scientists and clinical researchers.”

(Researcher #8, Clinician Investigator)

In addition to policy development, researchers felt they lacked knowledge about return of results in general and called for more education on the subject:

“… this is going to be more common practice as we are moving forward in this era of technology. So I think it's a good thing that we're looking at this, to figure out, should this be standardized? And, if so, who is standardizing it, you know, across the board? There's a lot of training as a research professional that you can have, you know, we have your CITI and your Good Clinical Practice. And this may be a whole other module.”

(Researcher #2, Recruitment/Consent)

## Discussion

**Fig. 1:**
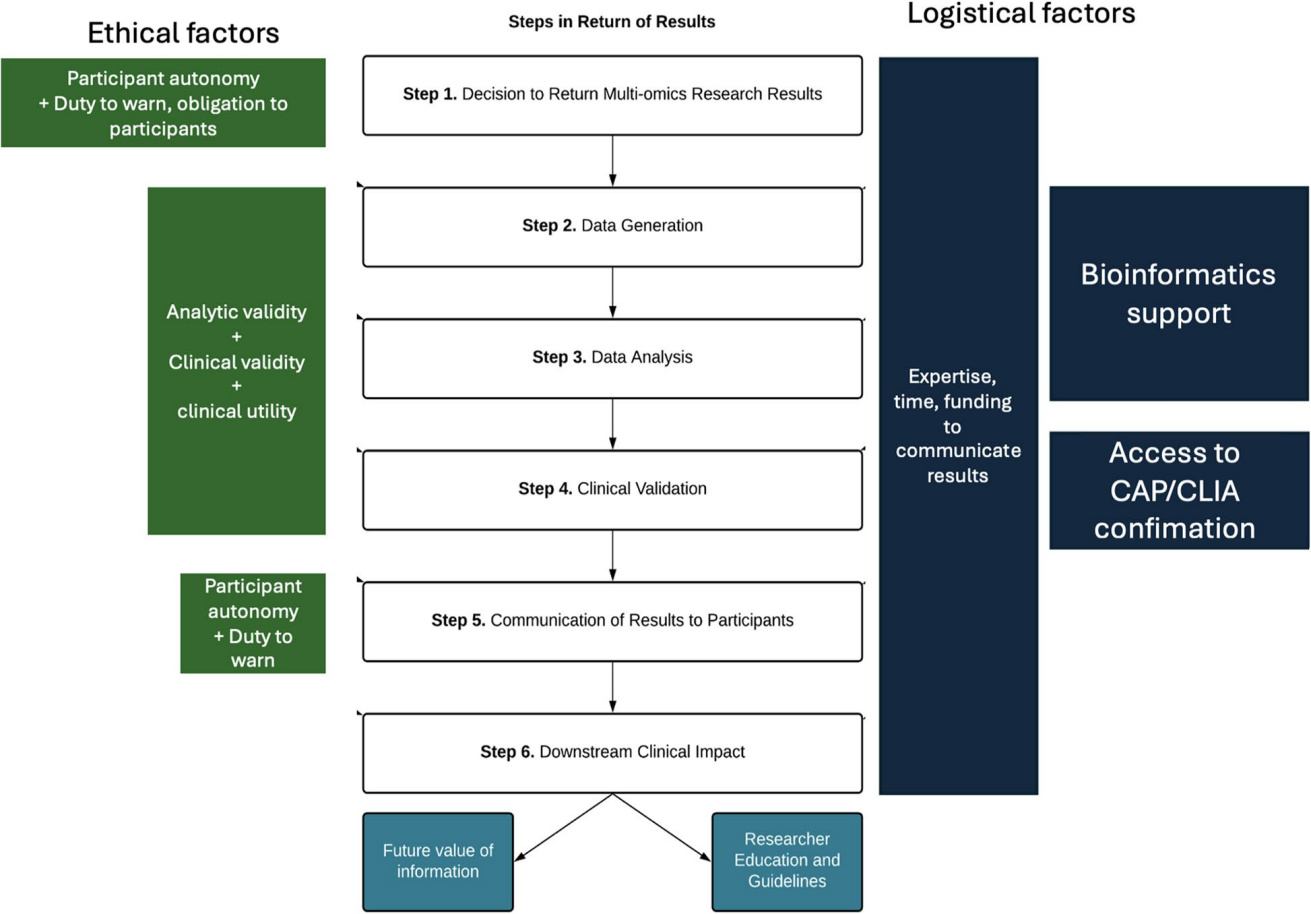
Ethical considerations (left; green), and logistical constraints (right; blue) from thematic analysis mapped onto steps in the return of results process. Future implications of returning results from multi-omics research shown at the bottom. Themes were shortened to simplify the diagram

We conducted semi-structured qualitative interviews to elicit researcher’s views regarding the individual return of multi-omic research results in the context of a specific study, MoTrPAC. The findings of this study represent unique perspectives about the return of multi-omics research results that have not been well described in existing literature. These factors and themes map to all steps in the return of results process (Fig. 1) including the decision to return results, data generation, data analysis, data validation, communication with participants, and downstream clinical impact.

Most researchers in this cohort expressed positive attitudes regarding the return of multi-omics results, particularly if they were considered actionable. This finding is consistent with researchers’ positive attitudes towards genomics return of results reported previously [28, 30, 43]. Respect for participant autonomy, perception of minimal harms and future value of multi-omics information were brought up to justify returning multi-omics results, and closely align with the ethical principles outlined by various expert working groups [2, 5, 44]. However, the positive attitudes reported should be interpreted in light of findings that suggested researchers who did not have clinical training (reflecting the majority of our participants – see Table 2), provide clinical care to research participants, or have prior experience returning research results were generally more inclined to offer return of results [45]. Furthermore, the depth of the relationship between researcher and participant, degree of dependence or vulnerability of the study population, and the importance of the reasons against return (e.g. costs to the research enterprise or uncertain benefit) may modify the relative strength of the ethical principles which justify return [5, 7].

While researchers in the present study were hypothetically supportive of returning multi-omics results, some questioned whether this was an appropriate use of resources given uncertain clinical validity and utility of data generated in the study. Researchers' perception of the clinical utility of multi-omics was informed by their knowledge of clinical testing practices and understanding of multi-omic data. Given the breadth of information generated, researchers stressed the importance of an informed consent process that effectively conveys which results have clearly established clinical utility and which do not. While researchers’ ethical concerns about return of multi-omics results mirror many of the early concerns for genomics return of results [2], our study participants were beginning to appreciate how the issues will play out for multi-omics specifically. It is notable, though, that researchers did not focus much on how privacy might be impacted in a way that was specific to multi-omics, but rather only mentioned that this has been a concern in genomics. We note concerns about the identifiability of multi-omic data in open-access databases have already emerged, given that RNA sequences and methylation patterns can predict DNA genotypes [46]. Current regulations under GINA may not fully cover these emerging vulnerabilities, necessitating updated privacy protections for multi-omic data [47, 48]. This is certainly an area that warrants more research, as many institutions are being asked to share multi-omics data broadly, often in an openly accessible manner.

Researchers in this cohort also expressed uncertainty regarding current policy and obligations, and felt more specific guidance was required for return of results protocols. These findings suggest there is a need for researcher education on return of results in general and the clinical utility of the multi-omics to ensure this key stakeholder group is able to make evidence-based decisions for implementation. Practical aspects of return of results such as time, funding, and limited access to genetic counselors are known to influence the decision of researchers or IRB members to return results [30, 49].While logistical barriers and costs are a common theme in return of results literature [2, 50], this work highlights the particular challenges researchers may perceive as barriers in the context of multi-omics—for example, researchers questioned if the infrastructure available could implement a return of results protocol, such as secure portals able to store large multi-omics data sets in a HIPAA compliant manner, funding, genetic counseling resources and follow up care. When analyzing multi-omic data sets that are multi-modal and represent dynamic biological processes, there are challenges with establishing gold standards for analysis and interpretation. Laboratory protocols to generate multi-omic data struggle with reproducibility (analytic validity), statistical methods to analyze data yield different results (analytic validity), and there is no consensus for what the spectrum of “normal” looks like with multi-omic (ex: a “normal” transcriptome profile) (clinical validity) [51–53]. These factors all complicate the ability to determine if a result is clinically useful or not.

While feasibility of return is currently an important question for return of results from multi-omics data, multiple large precision medicine studies are already returning medically actionable genetic sequencing results [54–59]. For example, in order to return results, the National Institute of Health (NIH)’s All of Us Research Project [57] established a grant funded genetic counseling resource (NIH award OT2 OD028251), which has to-date returned results to approximately 100,000 participants [60]. Other large precision medicine projects such as MyCode (Geisinger Health) integrate the return of results process for a set list of genes found in the research setting into study participants electronic medical record and inform the participant’s clinical care team of the research results [55, 61, 62]. These works on genomics return of results will inform relevant stakeholders to develop return of results consensus guidelines for other -omics in the future.

### Study limitations and future directions

While the researchers interviewed in this study are diverse in specialty, role, institution, and experience and represent views of multi-omic researchers, the majority of participants held non-clinical roles, had not personally returned genomic or multi-omic research results, and none of the researchers interviewed were clinical genetics providers. Since this study was conducted in 2020–2021, experience and attitudes towards multi-omic data may have changed in the intervening years. Finally, respondents to our recruitment survey may have been biased towards researchers with a strong opinion on return of results.

## Conclusions

Overall, researchers expressed positive attitudes toward the return of multi-omic research results in principle, citing participant rights to their own data and perception of minimal harm. However, competing ethical considerations, logistical constraints, and a need for more external guidance were raised as key concerns. We provide a roadmap (Fig. 1) for future researchers to consider as they design and implement processes around the return of individual research results from multi-omics data. Future studies should consider views of other relevant stakeholders: study participants, clinical genomics professionals, ethicists, policy makers, legal experts, to expand the knowledge base regarding the clinical utility of multi-omics information.

## Supplementary Information

Supplementary Material 1.

## Data Availability

De-identified transcripts, interview guides and other data are available upon reasonable request to the corresponding author.
