# Supplementary material for: Researcher views on returning results from multi-omics data to research participants: insights from The Molecular Transducers of Physical Activity Consortium (MoTrPAC) Study
Source: BMC Med Ethics. 2025 Feb 7;26:22. doi: 10.1186/s12910-025-01174-9 (PMC11804059; doi:10.1186/s12910-025-01174-9)
Supplement: Supplementary file 1 — Supplementary Material 1. [file 12910_2025_1174_MOESM1_ESM.pdf]

Appendix: Interview guide

Lets go ahead and get started. First, I'd like to learn a bit more about you and your role in MoTrPAC.	
**Questions & Notes**	**Triggers for probing**
**1. What do you do as part of the MoTrPAC team?**	- How long have you been involved in MoTrPAC?<br>- What is your training and background before MoTrPAC?<br>- What proportion of your time revolves around this project?
Thanks for sharing-- it really helps me to know more about you to guide the rest of our conversation. Next, I want to ask you about about the types of data collected and your understanding of the plans for returning results.	
**Questions & Notes**	**Triggers for probing**

**2. What is your understanding of the types of data/ analysis done as part of this study?**	- What omics?<br>- Physical measurements?<br>- Health history for the participant or the participant's family?<br>- How will this data be used?

**4. What do you know about the plans for returning individual research results*, generated in the study to participants?**	- Reminder to affirm if don't know about them and then follow up, why do you think that is the case?<br>- What is your understanding of the MoTrPAC consent process around returning individual research results to participants?<br>- What challenges do you see?<br>- What are the benefits?

Now, we've talked about the data generated in MoTrPAC and your understanding of the current plans to return it–thanks for sharing your feedback so far. Next, I'm going to ask about hypothetical results that could be generated from this study and your opinions on returning them. These results would be specific to each individual participant.	
**Questions & Notes**	**Triggers for probing**
**5. Some of these results could change participants medical care including the prevention, management or treatment of a disease or health condition. For example, participants could learn...**<br><br>**Do you think participants would be interested in that information?**<br><br>(National Academies Press, 2018; Waltz et al. 2017: Brothers et al. 2017)	- They have high levels of cholesterol. **Immediate treatment/medications might be recommended.**<br>- They are at increased risk for cancer in the future, some of which have prevention, recommended surveillance, or available treatments.<br>- They are at increased risk for a heart condition. It might mean they have one now or that they could develop one in the future.<br>- They may respond poorly to a specific medication, such as codeine or citalopram.

**6. What are your thoughts on receiving information that could uncover health risks relevant to other family members (like parents, brothers, sisters, children)?**	*How does that result differ from XX?*<br><br>*What is it about XX that makes you think participants wouldn't want to learn that?*<br><br>*How does high cholesterol compare to increased future cancer risk? etc*<br><br>- They are a carrier of a genetic condition that might put their current or future children at risk.<br><br>- Could be important for other families members medical management<br><br>- What are your thoughts on that?
**7. Do you think participants would be interested to receive results about conditions for which there are limited treatments or interventions? (**National Academies Press, 2018)	For example, participants could learn about increased risk for Alzheimer's or ALS/Lou Gerhig's disease.

**8. What are your thoughts on the returning to personalized results to participants that are investigational, meaning they may not be completely accurate or may be a false-positive-- research results ?**	**For example:**<br>- **Transcriptome-** BRCA1 mutation from RNA seq<br>- **Metabolome** profiles have been altered in common dz like obesity and diabetes, CHD, but also Alz Schizophrenia<br>- **Proteomics -** characterizing protein aggregation implicated in neurodegenerative diseases<br><br>a. What if there was no way to confirm the accuracy of that finding?<br>b. What if there was no way to rule out a potential false- positive?<br>c. Specifically ask about each -ome<br>i. Why or why not?

Last, I’d like to ask you a few questions about how the protocol for return of results should be developed.	
**Questions & Notes**	**Triggers for probing**
**8. Hypothetically, How would you determine which results, if any, participants should have the option to receive?**<br>(Jarvik et al., 2014; National Academies Press, 2018)	- What factors are important in determining which results if any should be returned? (usefulness/value to participant vs. burden on researchers)<br>a) Some people have discussed how results which are “**useful”** to participants should be returned, how would you define what is “useful to participants”?<br>- Personal health<br>· Family health<br>· Useful for future planning<br>· Personal curiosity<br>· Others?

**9. Personalized results could be shared a few different ways, such as online, over the phone, or from their doctor. How do you think personalized research results should be shared?** (Tabor et al., 2017)	b) How certain should we be in those results to return them?<br><br>- Who should have a say in what personalized results are returned to participants?<br><br>- Who should actually return those results?<br><br>- What do you think participants would do with these results once returned?
**10. Are there any other thoughts you would like to share about the return of results process for MoTrPAC?**	Probe to examine any issues mentioned
